# Amyloid Cardiomyopathy in Hereditary Transthyretin V30M Amyloidosis - Impact of Sex and Amyloid Fibril Composition

**DOI:** 10.1371/journal.pone.0143456

**Published:** 2015-11-23

**Authors:** Sandra Arvidsson, Björn Pilebro, Per Westermark, Per Lindqvist, Ole B. Suhr

**Affiliations:** 1 Department of Clinical Physiology, Heart Centre, Umeå University, Umeå, Sweden; 2 Department of Cardiology, Heart Centre, Umeå University, Umeå, Sweden; 3 Department of Public Health and Clinical Medicine, Umeå University, Umeå, Sweden; 4 Department of Immunology, Genetics and Pathology, Uppsala University, Uppsala, Sweden; 5 Department of Surgical and Perioperative Sciences, Umeå University, Umeå, Sweden; Consejo Superior de Investigaciones Cientificas, SPAIN

## Abstract

**Purpose:**

Transthyretin V30M (ATTR V30M) amyloidosis is a phenotypically diverse disease with symptoms ranging from predominant neuropathy to exclusive cardiac manifestations. The aims of this study were to determine the dispersion of the two types of fibrils found in Swedish ATTR V30M patients -Type A consisting of a mixture of truncated and full length ATTR fibrils and type B fibrils consisting of full length fibrils, and to estimate the severity of cardiac dysfunction in relation to fibril composition and sex.

**Material and Methods:**

Echocardiographic data were analysed in 107 Swedish ATTR V30M patients with their fibril composition determined as either type A or type B. Measurements of left ventricular (LV) dimensions and evaluation of systolic and diastolic function including speckle tracking derived strain were performed. Patients were grouped according to fibril type and sex. Multivariate linear regression was utilised to determine factors of significant impact on LV thickness.

**Results:**

There was no significant difference in proportions of the two types of fibrils between men and women. In patients with type A fibrils, women had significantly lower median septal (p = 0.007) and posterior wall thicknesses (p = 0.010), lower median LV mass indexed to height (p = 0.008), and higher septal strain (p = 0.037), as compared to males. These differences were not apparent in patients with type B fibrils. Multiple linear regression analysis revealed that fibril type, sex and age all had significant impact on LV septal thickness.

**Conclusion:**

This study demonstrates a clear difference between sexes in the severity of amyloid heart disease in ATTR V30M amyloidosis patients. Even though type A fibrils were associated with more advanced amyloid heart disease compared to type B, women with type A fibrils generally developed less cardiac infiltration than men. The differences may explain the better outcome for liver transplanted late-onset female patients compared to males.

## Introduction

Amyloid cardiomyopathy is frequently encountered in patients with transthyretin (ATTR) amyloidosis. It is especially prevalent in wild type ATTR amyloidosis (ATTRwt) where amyloid cardiomyopathy is the predominant manifestation. To date, more than 120 known amyloidogenic transthyretin (TTR) mutations are recognized, and commonly a mix of symptoms is presented that include sensory and motor neuropathy as well as cardiac manifestations [1]. Only a few TTR mutations are known to exclusively cause cardiac or pure neuropathic disease [1–3].

The phenotypic diversity is not only explicit between different TTR mutations but also described within mutations and even within families carrying the disease. This is the case for the TTR V30M mutation, found endemically in the northern parts of Sweden, and in regions in Portugal and Japan. Patients carrying the TTR V30M mutation commonly show a mixed phenotype but with substantial variation in disease manifestations and age at onset in different geographical locations [4]. Swedish ATTR V30M patients commonly have a late disease onset (mean age at onset 56 years) [5] in contrast to endemic areas in Japan and Portugal where onset of symptoms occurs earlier, usually in the fourth decade [6].

Moreover, in patients carrying the V30M mutation, male preponderance and predominantly late age of onset (>50 years) of disease have been described in those developing amyloidotic cardiomyopathy [7–9]. Intriguingly, ATTRwt amyloidosis almost exclusively affects elderly males [10, 11], and hormonal protection against amyloid heart disease has been suggested for women [12]. However, it has not been clearly elucidated what factors are involved in preventing females from developing ATTR cardiomyopathy to the same extent as males.

Recent studies have proposed biochemical differences in the composition of TTR fibrils as a possible explanation for phenotypical variation within the TTR V30M population. The amyloid fibrils are constituted either of a mix of full length and fragmented TTR (Type A) or only full length TTR (type B) [13]. Type A fibrils appear to be associated with development of increased myocardial thickness and are more commonly found in late-onset patients [14]. We have previously shown that patients with type A fibrils were more prone to develop ATTR cardiomyopathy post liver transplantation (LT) in comparison to patients with only type B fibrils [15]. Type B fibrils have mainly been detected in ATTR V30M patients whereas the mixed fibril type, type A, seems to be the standard in the majority of other TTR mutations, as well as in all patients with ATTRwt [13, 16].

The aims of the study were to determine the dispersion of the two types of fibrils in Swedish V30M patients, and to evaluate the frequency and severity of myocardial involvement in relation to sex and fibril composition.

## Patients and Methods

Clinical and echocardiographic data were analysed in 107 patients (72 males and 35 females) with tissue biopsy and genetically proven ATTR V30M amyloidosis. The data comprised all patients that had had their fibril type settled, along with an echocardiographic examination recorded digitally (i.e., from 2003 or later) at the University Hospital of Umeå, Sweden. Fourteen patients had undergone liver transplantation prior to the echocardiographic examination.

Patient files were thoroughly reviewed for patient history regarding hypertension, initial symptoms, disease duration, medical treatment, history of coronary artery disease and other severe cardiac disorders. Hypertension was defined as systolic blood pressure exceeding >140 mmHg or diastolic blood pressure exceeding 90 mmHg at repeated visits or ongoing hypertensive treatment. Initial symptoms were defined as patient reported symptom leading to health care contact. In the group with cardiac symptoms we also included patients diagnosed by echocardiographic findings of cardiomyopathy in the absence of other symptoms that could be attributed to ATTR amyloidosis. Thirteen patients were excluded from the echocardiographic evaluation due to comorbidities that were deemed to have profound impact on the outcome of the examination: previous cardiac surgery (n = 5), status post myocardial infarction (n = 1), severe aortic stenosis (n = 1), pacemaker rhythm (n = 4) and atrial fibrillation (n = 2).

### Tissue preparation and Western blot analysis

In brief, unfixed subcutaneous abdominal adipose tissue biopsies were cut into smaller pieces, washed in a 0.15 M NaCl solution containing 0.02% sodium azide, followed by lysis of erythrocytes by incubation in 0.88% ammonium chloride. Thereafter the material was defatted in several changes of acetone and left to dry in air [17]. The dried tissue samples were, as previously described, separated using Sodium Dodecyl Sulfate Polyacrylamide Gel Electrophoresis and analysed by western blot [14]. To detect full-length TTR and C-terminal TTR fragments, a polyclonal antiserum produced in rabbit against TTR50-127 was used [13, 14].

### Echocardiographic examination

Echocardiographic examination was performed in median 3 years (range 1–22) after onset of symptoms. Patients were investigated with two-dimensional, Doppler and M-Mode echocardiography (Vivid 7 and Vivid E9, GE Medical systems, Horten, Norway) using a phased-array transducer (1.5–4.0 MHz). Image acquisition was obtained from parasternal long axis and apical four- and two-chamber views. Pulsed and continuous Doppler flow velocities across the mitral valve and left ventricular (LV) outflow tract were acquired according to American Society of Echocardiography (ASE) [18]. Offline analysis was performed using commercially available software, Echopac PC, version 113 (GE Ultrasound, Horten, Norway).

From parasternal long axis view, LV diastolic dimensions (LVDD), interventricular septal thickness (IVST), and posterior wall thickness (PWT) were measured in end-diastole (Q-wave in the ECG). IVST >12 mm was noted and used as a marker for amyloid heart disease [19]. LV systolic diameter (LVSD) was measured in end-systole. Morphological measurements were all performed concordant to ASE guidelines [20]. In LV outflow tract (LVOT) the midsystolic distance between the aortic cusps was measured for calculation of LVOT area. From apical views, LV mass was calculated using the modified Devereux formula [21] and indexed to height (LVMI) [22]. LV ejection fraction (LVEF) was determined using Simpson biplane model. Left atrial volume (LAVI) was measured from using biplane area-length method and indexed to Body Surface Area (BSA). In order to assess diastolic function, early (E) to late (A) diastolic velocities ratio (E/A) and isovolumic relaxation time (IVRT) were measured from pulsed wave Doppler recordings at the mitral tips. By measuring the peak LV basal lateral early diastolic velocity (em) acquired from pulsed tissue Doppler recordings, E/em was calculated [23]. From pulsed wave Doppler recordings in the LV outflow tract and the use of LVOT area, stroke volume (SV) and cardiac output (CO) were calculated and indexed to BSA to obtain of stroke and cardiac index (SI and CI).

Speckle tracking analysis derived from B-mode apical four- and two-chamber images was performed to determine LV deformation or strain. The LV myocardium was manually outlined using a region of interest (ROI) and the software automatically defined segmental strain by dividing the LV into six segments in each echocardiographic view. Segmental strain values were averaged generating global LV longitudinal strain throughout the cardiac cycle. Global end-systolic LV strain and basal septal strain were measured using aortic valve closure from pulsed wave Doppler recordings of LV outflow tract as time landmark. Only segments deemed appropriate for analysis were accepted and a minimum of five accepted segments in each view was considered sufficient to continue with global strain analysis.

### Statistical analyses

Statistical analyses were performed using IBM SPSS Statistics, version 22 for Windows. Categorical data were summarized in frequencies and percentages and Fisher’s exact test was used for testing the equality of proportions between patient groups. Continuous variables were described using median values and interquartile range (25th-75th percentile) if not stated otherwise. As a subset of the continuous variables were skewed, tests for univariate differences between groups were performed using Mann Whitney U test.

IVST was transformed using the natural logarithm (ln) for regression analysis, because it had a slightly skewed distribution and to avoid violating the assumption of homoscedasticity in the multivariate analysis. However, for illustration purposes these data are shown on a conventional linear scale in figures. Simple linear regression was used to test for associations between ln IVST and age in both type A and type B fibril patients. Multivariate linear regression analysis was employed in order to determine the major predictors responsible for variation in ln IVST. Independent variables that were a priori considered to be important for the outcome were included in the model, rendering fibril type, sex, age, hypertension and disease duration as predictors. A p value <0.05 was considered statistically significant.

### Ethics statement

All parts of the investigation conforms to the principles outlined in the Declaration of Helsinki. The study was approved by the Regional Ethical Committee in Umeå and written informed consent has been obtained from patients.

## Results

### Patient characteristics

Clinical characteristics for male and female ATTR amyloidosis patients are presented in Table 1.

**Table 1:** Patient characteristics for men and women with transthyretin amyloidosis.

	Men (n = 72)	Women (n = 35)	p value
**Type A/Type B, n**	36/36	13/22	0.223
**Age at exam, median years (range)**			
**Type A**	68 (52–79)	74 (56–86)	0.079
**Type B**	54 (31–76)	59 (30–79)	0.197
**Disease duration, median years (range)**	2 (1–18)	4 (1–22)	**0.016**
**Initial symptoms, n (%)**			
**Neuropathy**	62 (86)	33 (97)	0.214
**GI symptoms**	5 (7)	2 (6)	1.000
**Cardiac manifestations**	5 (7)	0 (0)	0.170
**Ocular manifestations**	1 (1)	0 (0)	1.000
**Liver transplanted, n (%)**			
**Type A**	1 (3)	1 (8)	0.443
**Type B**	5 (14)	7 (32)	0.102
**Cardiac comorbidities, n (%)**	6 (8)	1 (3)	0.423
**Pacemaker, n (%)**	5 (7)	3 (9)	0.715
**Hypertension, n (%)**			
**Type A**	18 (50)	8 (61)	0.475
**Type B**	11 (31)	12 (55)	0.070

Type A, mixture of intact and fragmented transthyretin; Type B, only full length transthyretin. Continuous data are presented as median (range) and categorical data are presented as counts and percentages. Statistically significant differences are marked in bold.

Male to female ratio among type A patients was 36:13 and for type B patients 36:22. Median disease duration was significantly longer for women compared to men (p = 0.016) and among patients with type A fibrils, women tended to be older than males (p = 0.079). In patients with type B fibrils, hypertension tended to be more frequent in women, although this difference did not reach statistical significance (p = 0.070). Motor and/or sensory neuropathy were the most frequent initial symptoms for both sexes, commonly accompanied by findings of an increased myocardial thickness on the echocardiogram. Only five patients (all males) had exclusively symptoms related to cardiac manifestations at the diagnostic work-up.

### Cardiac involvement in patients with type A versus type B fibrils

**Fig 1:**
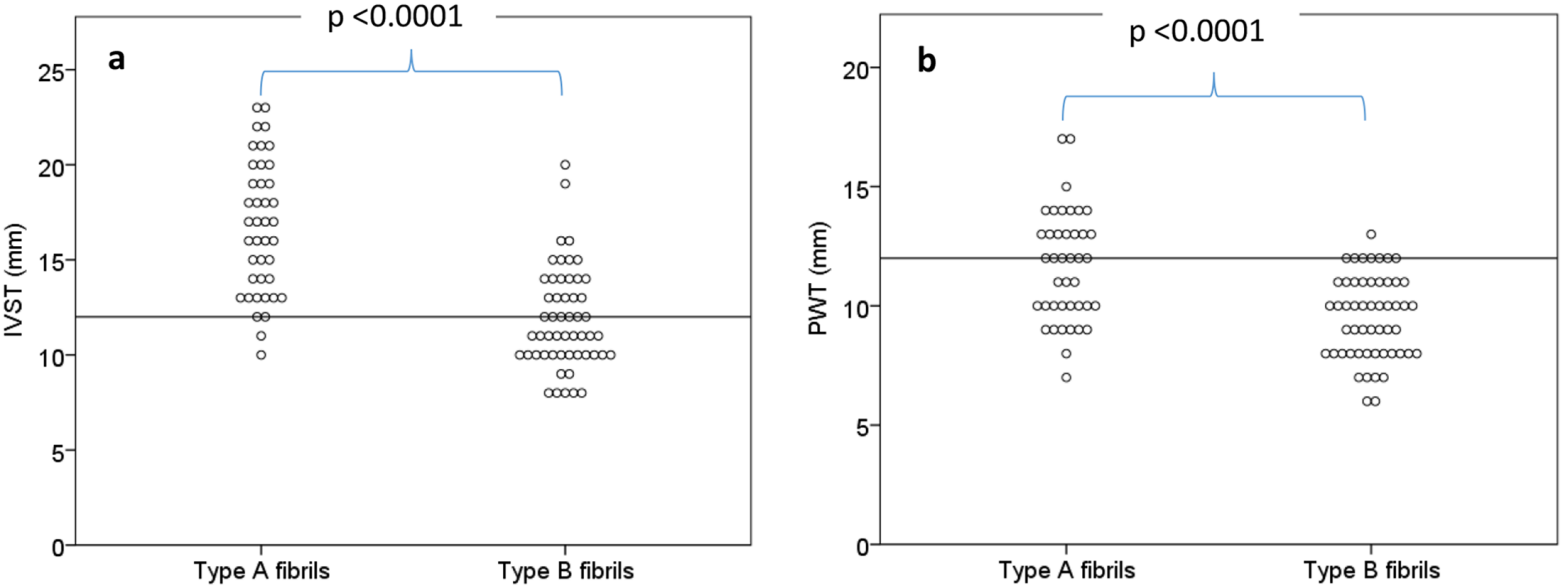
**(a)**. **Interventricular septal thickness in type A versus type B fibrils.** Horizontal line represents the limit for increased septal thickness (12 mm). **(b). Posterior wall thickness in type A versus type B fibrils**. Horizontal line represents the limit for increased posterior wall thickness (12 mm).

Patients with type A fibrils had more advanced amyloid heart disease than patients with type B fibrils. This was shown by significantly increased median IVST and PWT (p<0.0001) (Fig 1), higher E/em (p = 0.002) indicating higher frequency of increased filling pressures, and reduced LV systolic global strain (p = 0.005) in type A patients (S1 Table).

### Sex-related echocardiographic findings

A summary of the echocardiographic findings for patients grouped according to fibril type and sex are shown in Table 2.

**Table 2:** Echocardiographic findings for patients with transthyretin amyloidosis—comparison between men and women for the two respective types of fibrils.

Amyloid fibril composition	Type A			Type B		
	Men (n = 30)	Women (n = 11)	p value	Men (n = 33)	Women (n = 20)	p value
Heart rate, bpm	74 (64–82)	70 (66–80)	0.437	78 (65–88)	76 (65–87)	0.699
Pericardial effusion, n (%)	5 (17)	2 (18)	0.100	0 (0)	1 (5)	0.365
**Dimensions**						
IVST >12 mm, n (%)	29 (97)	8 (73)	0.052	14 (42)	5 (25)	0.247
IVST, mm	18 (15–20)	14 (12–16)	**0.007**	12 (10–15)	11 (10–13)	0.243
PWT, mm	12 (11–13)	10 (9–10)	**0.010**	10 (8–11)	9 (8–10)	0.054
LVDD, mm	49 (42–52)	47 (39–50)	0.329	48 (46–51)	47 (44–50)	0.467
LVSD, mm	29 (24–34)	28 (25–31)	0.657	29 (25–34)	28 (24–31)	0.424
LVMI, g/m	166 (135–209)	114 (108–152)	**0.008**	118 (90–130)	97 (81–121)	0.248
LAVI, ml/m^2^	33.0 (23.0–42.8)	27.8 (21.5–41.6)	0.528	24.1 (20.1–33.4)	21.8 (18.6–33.7)	0.678
LVEF, %	64 (58–69)	65 (58–74)	0.676	63 (58–71)	66 (57–71)	0.585
**Doppler measurements**						
E/A	0.8 (0.7–1.0)	0.9 (0.8–1.2)	0.346	1.1 (0.9–1.4)	0.9 (0.7–1.2)	0.126
E/em	10.0 (8.0–13.6)	9.3 (7.6–14.4)	0.957	6.4 (5.2–9.8)	8.6 (6.0–11.9)	**0.030**
IVRT, ms	94 (76–114)	95 (90–124)	0.788	80 (63–92)	83 (67–90)	0.545
SV, ml	78 (70–89)	73 (58–92)	0.546	76 (65–90)	72 (62–84)	0.202
SI, ml/m^2^	41 (37–45)	43 (35–55)	0.643	36 (34–47)	43 (36–49)	0.233
CO, l/min	5.9 (5.1–6.4)	5.5 (4.1–6.1)	0.192	5.7 (5.2–6.8)	5.2 (4.7–6.0)	**0.022**
CI, l/min/m^2^	3.1 (2.6–3.4)	3.2 (2.5–3.8)	0.797	2.9 (2.7–3.3)	3.1 (2.6–3.5)	0.707
**Speckle tracking derived longitudinal strain**						
Septal basal strain, %	-7.8 (-4.5–-11.8)	-11.9 (-8.8–-14.8)	**0.037**	-15.3 (-12.0–-17.6)	-16.4 (-13.3–-18.8)	0.270
LV global strain (a4c), %	-15.4 (-14.2–-17.7)	-18.0 (-15.6–-19.7)	0.057	-19.1 (-17.4–-20.1)	-19.1 (-15.8–-21.0)	0.924

*Data are presented as median (interquartile range)*, *unless stated otherwise*. *Type A*, *amyloid fibril composed of a mixture of full length and fragmented transthyretin; Type B*, *full length transthyretin only; IVST*, *interventricular septal thickness; PWT*, *posterior wall thickness; LVDD*, *left ventricular diastolic diameter; LVSD*, *left ventricular systolic diameter; LVMI*, *Left ventricular mass index; LAVI*, *left atrial volume index; LVEF*, *left ventricular ejection fraction; E/A; early/late mitral diastolic filling velocity; E/em*, *early mitral diastolic filling/early myocardial diastolic filling velocity; IVRT*, *Isovolumic relaxation time; SV*, *stroke volume; SI*, *stroke index; CO*, *cardiac output; CI*, *cardiac index*. Statistically significant differences are marked in bold.

**Fig 2:**
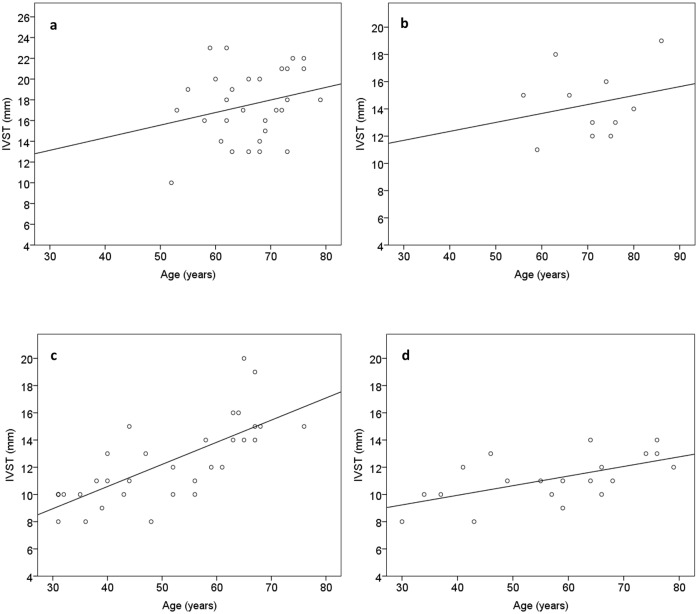
Scatterplot of interventricular septal thickness (IVST) in relation to age in. a) male patients with type A fibrils. The regression equation for ln IVST = 2.32+0.008*age, R^2^ = 0.073, p = 0.148. b) female patients with type A fibrils. The regression equation for ln IVST = 2.34+0.004*age, R^2^ = 0.052, p = 0.501. c) male patients with type B fibrils. The regression equation for ln IVST = 1.82+0.01*age, R^2^ = 0.563, p<0.0001. d) female patients with type B fibrils. The regression equation for ln IVST = 2.02+0.007*age, R^2^ = 0.364, p = 0.005.

Virtually all males (97%) and the majority of women (73%) carrying type A fibrils had increased IVST (> 12 mm), with borderline higher proportions for males (p = 0.052). Among patients with type B fibrils, increased IVST was noted in 42% of males and 25% of females. For patients with type A fibrils, men demonstrated significantly higher median values for the morphologic descriptors IVST (p = 0.007), PWT (p = 0.010) and LVMI (p = 0.008). Male patients also displayed reduced LV longitudinal strain as compared to women, this difference was especially pronounced for basal septal strain (p = 0.037) and borderline significant for global LV longitudinal strain (p = 0.057). Among patients with type B fibrils, lower E/em (p = 0.030) and higher CO (p = 0.022) were found in males as compared to females. Simple linear regression analysis revealed a positive association between age and ln IVST in both women (p = 0.005) and men (p<0.0001) with type B fibrils. This relationship was not present for either sex in patients with type A fibrils (Fig 2).

Table 3 presents the results of the multiple regression analysis with explanatory factors to ln IVST.

**Table 3:** Results of multiple regression analysis for factors associated with the natural log of left ventricular septal thickness (dependent variable).

	Regression coefficient B	Std. Error	Standardized Beta	p value
(Constant)	1,817	0.098		<0.0001
Fibril type	0.203	0.044	0.369	<0.0001
Sex	0.202	0.040	0.350	<0.0001
Hypertension	0.073	0.041	0.132	0.077
Age (years)	0.008	0.002	0.424	<0.0001
Disease duration	0.008	0.005	0.109	0.113

Age, fibril type and sex were the major determinants for IVST. The multiple regression analysis revealed that fibril type (B = 0.203, CI 0.115–0.290), age (B = 0.008, CI 0.005–0.012) and sex (B = 0.202, CI 0.123–0.282) all had a positive association on ln IVST, whereas presence of hypertension and disease duration had no significant impact.

## Discussion

To our knowledge, this is the first study investigating ATTR cardiomyopathy in ATTR V30M amyloidosis patients and its relationship with sex and fibril composition. In this material, amyloid fibril composition, together with sex and age were the most important factors for development of cardiac amyloidosis. Patients with type A fibrils appear to suffer more severe cardiac involvement, irrespective of age, a finding that is concordant with earlier studies on smaller cohorts of Swedish and Japanese ATTR V30M patients [14–16]. We found no significant difference in fibril composition between the sexes.

A male predisposition for development of amyloid heart disease has been described in non-endemic late-onset (>50 years of age) ATTR V30M patients in Japan [24], in late-onset Swedish ATTR V30M patients [7, 25], and also for other TTR mutations [26, 27]. Furthermore, survival after liver transplantation is longer for late-onset females as compared to males [28]. In a study including several TTR mutations Rapezzi et al [12] described less myocardial involvement in younger women than in males, but no difference in patients of postmenopausal age. Similar to the cohort of Rapezzi et al, the majority of women with type A fibrils in our study were of postmenopausal age and virtually all presented with echocardiographic signs of amyloid myocardial infiltration. However, even in this group of late-onset females with type A fibrils, a less severe cardiac amyloid disease was noted compared with that of type A males. This sex-related discrepancy was not displayed among type B patients.

It is today well known that healthy females have lower wall thickness and LV mass than males [29]. Furthermore, females are more prone to develop heart failure due to hypertension and are more commonly diagnosed with heart failure with preserved ejection fraction [30]. Females have been shown to better preserve myocardial mass, myocyte diameter and volume, whereas loss of myocytes and reactive hypertrophy are more common in males [31]. Therefore, remodelling probably differs between the sexes, a difference that may be related to female oestrogen production [32]. Indexing volumes and LV mass by height or BSA does not completely eliminate the differences between sexes. However, in the present study, women with type A fibrils did not only have lower LV thickness and mass as compared to males, but also displayed more preserved systolic function. Thus, the demonstrated differences in LVMI between men and women with type A fibrils could not solely be attributed to general physiological sex discrepancies.

Interestingly, in an examination of amyloid content and variation between wild type and variant TTR in autopsy heart tissues Tasaki et al [33] found that the proportion of wild type ATTR in cardiac tissue positively correlated with increased age for both sexes, but more evident in men, whereas the total amount of amyloid increased with age for men but decreased for women. Amyloid fibril composition was not determined in the study, and since the age dependent increase was found in a cross-sectional study according to age at autopsy the observed increase may be population related, i.e., related to amyloid fibril composition where type A amyloid fibrils appear to be more prone for additional deposition of wild type ATTR [34]. However, the decreased amount of total ATTR noted for females with advancing age in the study by Tasaki et al. was not replicated by our study where type B patients’ IVST increased with age for both sexes. Whether the age correlated increase in IVST for type B patients mainly is related to continuous amyloid infiltration is unclear as the exact effect of type B fibrils on the heart is not completely known.

The phenotypic similarities between male patients with type A fibrils and patients with ATTRwt are immense, and it has been suggested that ATTR amyloidosis might evolve into ATTRwt disease in elderly males. Currently, we are reluctant to perform liver transplantation on especially male patients with type A fibrils [15, 25]. The finding of better outcome for late-onset female patients may well be explained by less cardiac involvement in women carrying type A fibrils as compared to males.

### Limitations

Patients with predominant or exclusive amyloid cardiomyopathy are likely to have a longer disease duration than perceived from onset of symptoms, this especially concerns patients with type A fibrils and a bias towards underestimated disease duration in those patient groups is probably present. Detecting the C terminal fragments in western blot analysis is impossible if the tissue sample only contains small amount of amyloid since the band corresponding to full length and fragment will be weak. In the present investigation, only samples with a clear band corresponding to fragment were denoted type A, thus, small amounts of fragments in biopsy tissues may be missed, and patients may be misclassified as type B patients.

Determination of amyloid fibril composition and echocardiographic examination were often not performed simultaneously. However, so far, no patients in whom repeated biopsies have been performed, have changed their type of amyloid fibril composition [34].

### Conclusion

This study demonstrates a clear difference between sexes in the severity of amyloid heart disease in ATTR V30M amyloidosis patients that is not related to fibril composition. Even though type A fibrils were associated with more advanced amyloid heart disease, women with type A fibrils generally developed less cardiac infiltration, as shown by thinner LV myocardial thickness and more preserved septal LV strain. The differences may explain the better outcome for liver transplanted late-onset female patients compared to males, and the impact of fibril composition and sex deserves to be explored for other treatment modalities.

## Supporting Information

S1 Table Echocardiographic characteristics in type A and type B transthyretin amyloidosis patients. Data are presented as median (interquartile range). Type A, amyloid fibril composed of a mixture of full length and fragmented transthyretin; Type B, full length transthyretin only; IVST, interventricular septal thickness; PWT, posterior wall thickness; E/em, early mitral diastolic filling/early myocardial diastolic filling velocity; LV, left ventricular.
(DOCX)
