# Supplementary material for: Amyloid Cardiomyopathy in Hereditary Transthyretin V30M Amyloidosis - Impact of Sex and Amyloid Fibril Composition
Source: PLoS One. 2015 Nov 23;10(11):e0143456. doi: 10.1371/journal.pone.0143456 (PMC4658178; doi:10.1371/journal.pone.0143456)
Supplement: S1 Table — Data are presented as median (interquartile range). Type A, amyloid fibril composed of a mixture of full length and fragmented transthyretin; Type B, full length transthyretin only; IVST, interventricular septal thickness; PWT, posterior wall thickness; E/em, early mitral diastolic filling/early myocardial diastolic filling velocity; LV, left ventricular. (DOCX) [file pone.0143456.s001.docx]

| **Echocardiographic characteristics in type A and type B transthyretin amyloidosis patients** | | | |
| --- | --- | --- | --- |
|  | Type A | Type B | p value |
| IVST, mm | 17 (14-20) | 11 (10-14) | <0.0001 |
| PWT, mm | 12 (10-13) | 10 (8-11) | <0.0001 |
| E/em | 10.0 (7.9-13.3) | 7.0 (5.5-10.4) | 0.002 |
| LV global strain (a4c), % | -15.8 (-14.3- -18.5) | -19.1 (-16.9- -20.5) | 0.005 |
| Data are presented as median (interquartile range). Type A, amyloid fibril composed of a mixture of full length and fragmented transthyretin; Type B, full length transthyretin only; IVST, interventricular septal thickness; PWT, posterior wall thickness; E/em, early mitral diastolic filling/early myocardial diastolic filling velocity; LV, left ventricular. | | | |

**S1 Table**
